# Recent advances in primary cilia in bone metabolism

**DOI:** 10.3389/fendo.2023.1259650

**Published:** 2023-10-10

**Authors:** Fenfen Lian, Hui Li, Yuwei Ma, Rui Zhou, Wei Wu

**Affiliations:** ^1^ School of Exercise and Health, Shanghai University of Sport, Shanghai, China; ^2^ School of Athletic Performance, Shanghai University of Sport, Shanghai, China

**Keywords:** primary cilia, bone formation, chondrogenesis, bone resorption, fracture healing, integrins

## Abstract

Primary cilia are microtubule-based organelles that are widespread on the cell surface and play a key role in tissue development and homeostasis by sensing and transducing various signaling pathways. The process of intraflagellar transport (IFT), which is propelled by kinesin and dynein motors, plays a crucial role in the formation and functionality of cilia. Abnormalities in the cilia or ciliary transport system often cause a range of clinical conditions collectively known as ciliopathies, which include polydactyly, short ribs, scoliosis, thoracic stenosis and many abnormalities in the bones and cartilage. In this review, we summarize recent findings on the role of primary cilia and ciliary transport systems in bone development, we describe the role of cilia in bone formation, cartilage development and bone resorption, and we summarize advances in the study of primary cilia in fracture healing. In addition, the recent discovery of crosstalk between integrins and primary cilia provides new insights into how primary cilia affect bone.

## 1 Introduction

Cilia are highly conserved organelles distributed on the cell surface and present in most cells of the human body (1). The cilium consists of a basal body and an axoneme (2), and its formation is mainly carried out through a transport mechanism that is completely homologous to that of Chlamydomonas reinhardtii and IFT proteins (3) which is closely related to the cell cycle (4), and it is a complex process that involves the assembly of microtubule structures and protein transport (5), including the fusion of centromeres with the plasma membrane (3) ciliary secretion (6), and the transport of Golgi vesicles (7). Early studies suggested that there are two modes of ciliary axoneme formation, namely nine double microtubules surrounding a central pair of single microtubules (9 + 2); and a central pair of microtubules missing (9 + 0) mode, due to the lack of axonemal dynamo proteins responsible for ciliary motility in 9 + 0 cilia are considered immotile and are also referred to as primary cilia (8).

However, with the in-depth study of primary cilia, some studies have found that the three-dimensional structural model of primary cilia established by cryo-cryo-tomography reveals that the structure of primary cilia is different from that of the “9 + 0” pattern (9), including a recent study that found that the “9 + 0” pattern arrangement exists only in the matrix of the primary cilium and that the structure of primary cilium changes to unstructured bundles of EB1-decorated microtubules and actin filaments just a few micrometers away from the matrix (10). Primary cilia contain hundreds and thousands of proteins, but interestingly primary cilia do not synthesize any proteins themselves. Ciliary proteins are mainly dependent on the intraflagellar transport system (IFT) for transport to the cilium. IFT is a bidirectional transport system operated by IFT protein complexes (IFT complex A, IFT complex B) and IFT motors, which play an important role in cilia assembly and maintenance (11, 12). IFT is regulated by the transition zone (TZ) at the base of the cilia (13), and during cilia assembly activates kinesin-II to transport large protein complexes from the cytosol to the tip of the cilium, the transported proteins are dissociated and released, and then kinesin-II is inactivated, kinesin-II is activated and binds to the IFT protein complex a, and retrograde movement towards the base of the cilium (4). Also, IFT is required to mediate many signaling functions (14) IFT mediates Hedgehog, Wnt, PDGFR, Notch, TGF-β, mTOR and other signaling pathways have been reported in detail in other reviews (4, 15, 16).

A large body of data shows that primary cilia play a crucial role in vertebrate development and human genetic diseases by regulating a variety of fundamental processes that contribute to the maintenance of cellular activity and tissue homeostasis, including cell differentiation and vertebrate development, cell cycle control, and signaling (17, 18). IFT is capable of initiating signaling pathways linking mechanical or chemical stimuli to intracellular transduction cascades that act at various sites in the body (19, 20). Many human diseases are associated with defects in ciliary structure or localized proteins in cilia (21). In recent years, it has been found that ciliopathy symptoms often involve skeletal development, highlighting the importance of primary cilia in skeletal development. This review summarizes recent findings on the role of primary cilia and the ciliary transport system in osteogenesis and development, We describe the role of cilia in bone formation, cartilage development, and bone resorption, and we also summarize advances in the study of primary cilia in fracture healing.

## 2 The role of primary cilia in bone metabolism

### 2.1 The role of primary cilia in bone formation

The skeletal system is a dynamic and highly coordinated system that involves a balance between bone formation by osteoblasts and bone resorption by osteoclasts (22). Bone formation is predominantly executed by osteoblasts (23), and primary cilia have been shown to have an important role in osteogenesis (24–26). It has been shown that primary cilia regulate the osteogenic differentiation of bone marrow-derived mesenchymal stem cells (27) and adipose-derived stem cells (28), and that they are involved in the process of osteoblast alignment and differentiation (29), regulating skeletal development in the embryonic period and bone formation in adulthood (30), and that knockdown of primary cilia reduces load-induced bone formation (31). In recent years, the role of pulsed electromagnetic fields in promoting bone formation has been gaining attention, however, primary cilia play a key role in this. It has been shown that primary cilia act as sensory organs involved in pulsed electromagnetic field-mediated bone formation (32). He (33) found that PEMF stimulates osteogenic differentiation mainly by activating the NOS/NO/sGC/cGMP/PKG signaling pathway and thus stimulating osteogenic differentiation, and it is noteworthy that all components of this signaling pathway, including iNOS, eNOS, sGC, PKG-1, and PKG-2 are localized in the primary cilia, and that eNOS needs to be phosphorylated within the primary cilia in order to be phosphorylated. As well as Liu (25) also reported that the primary cilia of osteoblasts in rats exposed to microgravity rapidly became shorter or even disappeared, and this phenomenon was accompanied by a significant reduction in osteogenic differentiation and mineralization of osteoblasts. In addition, it has been shown that cellular function can barely be maintained under conditions without mechanical loading and the effect of inducing osteoblasts to become bone cells is greatly limited (34), whereas primary cilia, as an important part of mechanotransduction, are involved in mechanosensing and signaling that regulates the behavior of mesenchymal bone progenitors, osteoblasts and osteoclasts (35).

The IFT protein family also plays an important role in bone formation (36), and the knockdown of IFT proteins leads to a reduction in mechanically stimulated paracrine signaling that promotes osteogenesis and affects osteogenesis (37). Guleria (23)found that silencing of IFT52 disrupts primary ciliogenesis preventing normal osteogenic differentiation. IFT52 silencing resulted in a significant decrease in mRNA levels of runt-related transcription factor 2 (Runx2), secreted phosphoprotein 1 (Spp1) and bone gamma carboxyglutamate protein (Bglap). the decrease in Patched-1 (Ptch1) demonstrated that silencing of Ift52 attenuates the upregulation of this microsignaling pathway (Hh). IFT140 as a core protein of IFT complex A has been shown to be closely associated with bone formation marker expression during differentiation of BMMSCs (38). Tao (39) established the Osx-Cre; Ift140flox/flox mouse model and found that the mice exhibited dwarf phenotypes such as short bone length, low bone mass and reduced bone mineral deposition rate as well as reduced expression of osteoblast markers. These results suggest that IFT140 is indispensable in the process of bone formation. Interestingly, IFT140 is temporally and spatially specific during bone formation, with the current study indicating that IFT140 mainly acts during the early stages of osteoblast differentiation (40). In addition, the differentiation programs of adipogenesis and osteoclastogenesis are in competition with each other and balance each other, with mechanisms that promote adipogenesis inhibiting osteoclastogenesis (41). A recent study demonstrated that IFT20 controls MSCs allocation by regulating glucose metabolism during skeletal development. IFT20 deficiency in MSCs promotes adipocyte formation, which enhances RANKL expression and reduces bone formation. Mechanistically, IFT20 deletion in MSCs reduces glucose tolerance, thereby inhibiting glucose uptake, lactate and ATP production. In addition, the deletion of IFT20 significantly reduced the activity of TGF-β-Smad2/3 signaling and decreased the binding activity of Smad2/3 to the Glut1 promoter, thereby downregulating Glut1 expression. These results suggest that IFT20 plays an important role in the balance between osteogenesis and adipogenesis through the TGF-β-Smad2/3-Glut1 axis (42). In addition, numerous reports of Ift80 and Ift88 being ablated in developing mouse limbs lead to defects in skeletal pattern formation (43–45), as well as a large number of skeletal ciliopathies in which mutations have been found on IFT43, IFT121, IFT122, and IFT80, IFT52, IFT172, and IFT56 (46).

In addition, it was found that polycystic kidney disease (PKD) gene product polycystin-1 (PC1), which is localized to primary cilia, can regulate bone development and osteoblast function through the regulation of the osteogenic transcription factor Runx2-II, and modulates intracellular calcium-dependent signaling in osteoblasts, and that depletion of intracellular calcium blocks PC1 activation of the Runx2-II P1, demonstrating the importance of the intracellular calcium pathway in PC1-mediated Runx2-II P1 promoter activity in osteoblasts. It is suggested that the polycystin complex in bone functions as an intracellular pathway that senses external developmental signals and translates them into the regulation of Runx2-II expression during bone formation (47). Moreover, Xiao (48) found that Pkd1 has a mechanotransduction role in bone, and conditional deletion of Pkd1 in osteoblasts revealed the reductions of bone mineral density, bone trabecular volume, and expression of Runx2, Osteocalcin, and Dmp1 in Dmp1-Cre; Pkd1flox/+, and Pkd1^Dmp1-cKO^ mice have all correlated with the Pkd1 gene dosage. Furthermore, Pkd1-deficient osteoblasts showed a markedly diminished response to fluid shear stress *in vitro*. These results suggest that Pkd1 has a direct mechanosensing role in osteoblasts to regulate osteoclast function and skeletal homeostasis. As well, studies have reported that silencing Pkd2 in osteoblasts also leads to reduced Runx2 expression and impaired biomechanical properties in mouse bone, thereby affecting bone formation. Interestingly, deletion of Pkd2 also led to reduced PPARγ expression, resulting in reduced bone marrow fat *in vivo* (49). These results suggest that Pkd1 and Pkd2 have synergistic roles in osteoblast differentiation and opposite roles in adipogenesis, and we therefore suggest that the Pkd1 and Pkd2 signaling pathways can independently influence the mesenchymal spectrum of bone (50, 51). Qiu (52) explored the mechanism and found that the expression of Gli2, a gene downstream of the Hh signaling pathway, and Axin2, a gene downstream of Wnt/β-catenin, were significantly reduced in osteoblasts of Kif3aOc-cKO mice compared with Kif3aflox/+ mice, and these results indicate that Kif3a deficiency affects the transduction of signaling pathways, such as Hh and Wnt/β-catenin, which in turn affects bone formation. However, some studies have found that the effect of Kif3a on osteoblasts may be mediated through polycystins (53), which needs to be further investigated.

We summarize recent relevant studies exploring the role of IFT proteins in bone formation (**Table 1**).

**Table 1:** Intraflagellar transport in bone formation.

Name	Study	Ref
Ift52	Silencing of Ift52 impaired primary ciliogenesis and hindered osteogenic differentiation. Up-regulation of the Hedgehog (Hh) pathway during osteogenesis is attenuated in Ift52-silenced cells	(23)
IFT140	Osx-Cre; Ift140flox/flox mice exhibit pygmy phenotypes such as short bone length, low bone mass and reduced bone mineral deposition rate and reduced expression of osteoblast markers.	(39)
Pkd1/PC1	Deletion of Pkd1 in Mature Osteoblasts, Reduced Expression of Runx2-II, Osteocalcin, Dmp1 and Phex in Bone of Pkd1flox/+ and Pkd1Dmp1-cKO Mice.	(48)
Pkd2/PC2	Silencing of Pkd2, Oc-Cre;Pkd2flox/null (Pkd2Oc-cKO) mice exhibit reduced bone mineral density, trabecular volume, cortical thickness, mineral deposition rate, and impaired biomechanical properties of bone.Pkd2 deficiency leads to reduced Runx2 expression in bone and affects osteoblast differentiation *in vivo*.	(49)
Kif3a	Colα1(I) 2.3-Cre;Kif3afl/fl mice have reduced new bone formation under mechanical ulnar loading.	(51)
	Kif3aOc-cKO mice developed osteoporosis at 6 weeks of age, as evidenced by reduced femoral mineral density, trabecular volume, and cortical thickness. Shh-mediated expression of Gli2 as well as Wnt3a-mediated expression of β-catenin and Axin2 were attenuated.	(52)
Ift20	Using Osterix-Cre and inducible type I collagen-CreERT, it was found that deletion of IFT20 in osteoblast cell lines resulted in impaired cell alignment and reduced bone mass. The results indicate that IFT20 regulates polarity and cell alignment during bone development through ceramide-pPKCζ-β-catenin signaling	(54)
IFT46	Knockdown of IFT46 results in the absence of cilia in different tissues.IFT46 morphants exhibit axial shortening, defective neural tube closure, and abnormal craniofacial development.	(55)
SAC	PEMF stimulation promoted intracellular sAC expression and increased cAMP concentration in hypoxia-exposed primary osteoblasts. Blockade of intracellular sAC inhibited the PEMF-induced decrease in HIF-1 α expression and increase in osteoblast differentiation.	(56)

**Figure 1:**
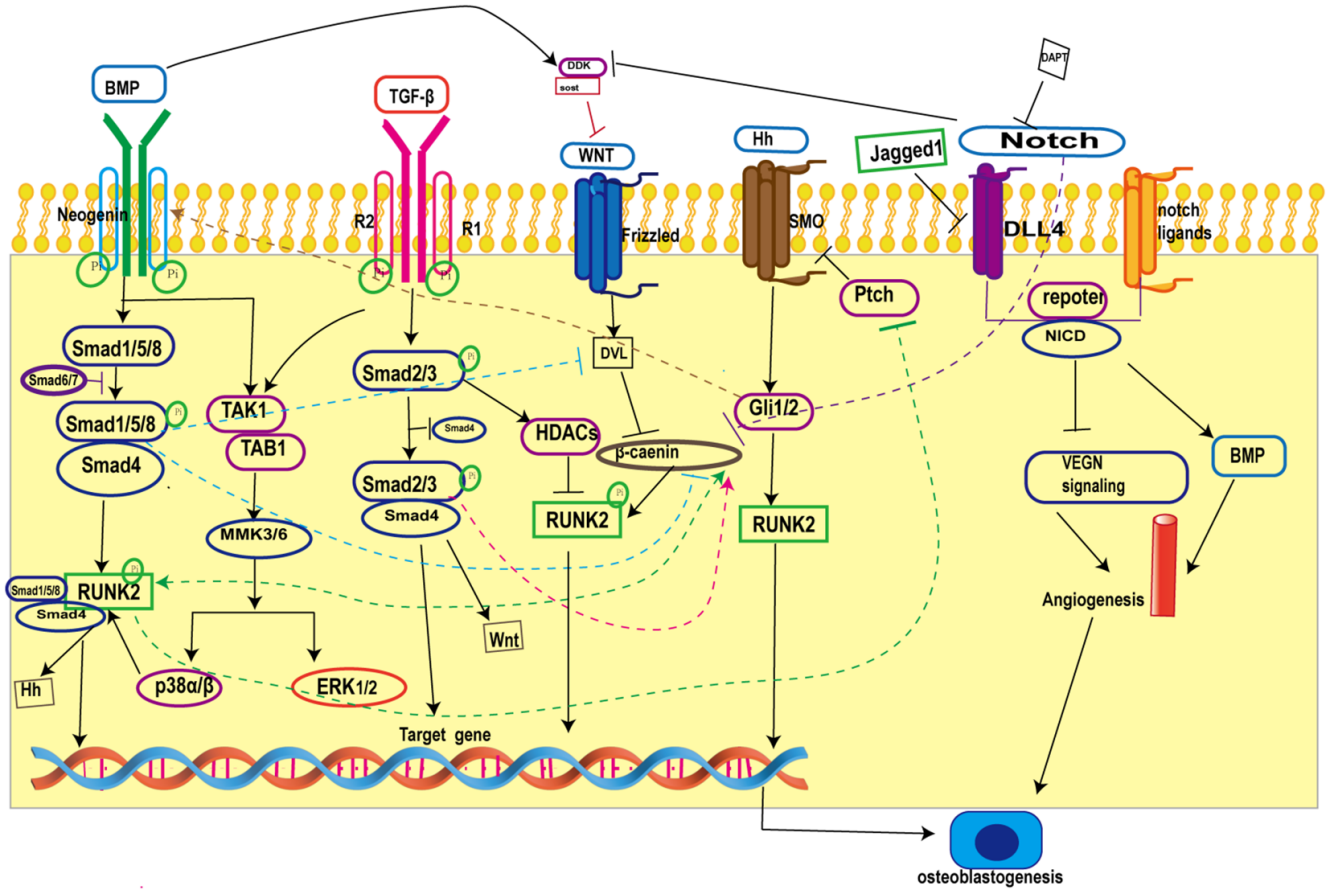
Crosstalk in primary cilia-related signaling pathways during osteogenesis. BMP inhibits Wnt/β-catenin signaling by increasing the expression of the Wnt antagonists Dkk1 and Sost and blocking β-catenin nuclear translocation; on the other hand, BMP promotes Wnt/β-catenin signaling by forming a co-transcriptional complex with β-catenin/Runx2 and antagonizing the function of Dvl by increasing the expression of Wnt. TGF-β promotes Wnt activity by **i**ncreasing Wnt ligand expression. Hh signaling promotes BMP signaling by enhancing BMP-2 expression through Gli. In addition, Notch intracellular structural domain (NICD1) enhances BMP-induced angiogenesis and thus promotes bone formation. BMP, Bone Morphogenetic Proteins; Runk2, Recombinant Runt Related Transcription Factor 2; TGF-β, transforming growth factor-β; ERK1/2, extracellular regulated protein kinases; Hh, Hedgehog; SMO, Smoothene.

Skeletal tissue development in mammals is complex and involves multiple signaling pathways interacting with each other, Primary cilia on osteoblasts are the arena for the confluence of many signaling pathways (17), and the interactions between primary cilia-related signaling pathways during osteogenesis are provided in the figure (**Figure 1**).

### 2.2 The role of primary cilia in bone resorption

Osteoclasts (OCs) are the cells responsible for physiological bone resorption and play an important role in maintaining bone mass homeostasis in the body (57). The current study indicates that osteoclasts derived from the hematopoietic lineage do not have primary cilia (58), but nevertheless, primary cilia are essential for their occurrence. PEMFs were found to inhibit osteoclast differentiation by scavenging reactive oxygen species (59) and modulating the Akt/mTOR signaling pathway (60). Wang (61)found that PEMFs affect bone resorption mainly by regulating RANKL/OPG to inhibit osteoclast formation, and this process is closely related to primary cilia, and its deletion of primary cilia was found to significantly enhance osteoclast formation and bone resorption. Parathyroid hormone (PTH) receptor 1 (PTH1R) is a B-group G protein-coupled receptor (GPCR), which plays an important role in bone metabolism, and activation of PTH1R was found to regulate osteoclasts. Tirado-Cabrera (62) reported the localization of PTH1R to primary cilia, which confirmed that primary cilia in osteoblasts are necessary for proper communication with osteoclasts.IL-6 secretion is increased when PTH1R is silenced. Previous studies have shown that IL-6 enhances osteoblast-mediated osteoclastogenesis by promoting the activities of JAK2 and RANKL (63). IFT80 can promote tumor necrosis factor (TNF) receptor-associated factor 6 (TRAF6) proteasomal degradation through binding to Casitas B-lineage lymphoma proto-ogene-b (Concbl-b), which regulates OC formation. Knockdown of IFT80 leads to Cbl-b ubiquitination and elevated TRAF6 levels, which overactivated the nuclear factor κβ (NF-κβ) receptor activator (RANKL) signaling axis (RANK/RANKL), thereby increasing OC formation, while IFT80 overexpression significantly inhibits OC formation and the downstream signaling pathway of RANKL/RANK (64).

### 2.3 The role of primary cilia in cartilage development and repair

Chondrogenesis denotes an intricately intricate process (65). While the importance of primary cilia in the progression of cartilage has been previously examined (66), recent investigations have furnished novel evidence regarding certain factors localized to primary cilia, including THM2, PTHrP, GRK2, and Gpr161, which participate in the development of cartilage (**Table 2**).

**Table 2:** The role of proteins or signalling molecules on primary cilia in cartilage development.

Name	Study related to cartilage development	Ref
THM2	The small phenotype of Thm2-/-, Thm1aln/+ mouse mutants is accompanied by structural defects in the small mandible and long bones (tibia) and decreased bone mineral density.	(46)
PTHrP	PTHrP chondrocytes are maintained in a Wnt-inhibited environment in the resting zone of the growth plate and regulate the formation, expansion and differentiation of chondrocytes in the resting zone.	(67)
GRK2	GRK2 deficiency leads to ATD through defects in ciliary signaling; GRK2 is a positive regulator of Hh signaling, and its absence leads to inhibition of GLI3 activity. inactivation of GRK2 leads to defects in cilia accumulation in SMO of chondrocytes as well as impaired Wnt signaling typical of GRK2-/- cells.	(68)
crocc2	crocc2 affects chondrocyte ability to sense and respond to the mechanical environment, with downstream effects on cartilage morphogenesis.	(69)
FGF2/FGF8	FGF2 and FGF8 are important growth factors for the growth of mandibular condylar cartilage in young mice and have a limited role in cartilage of aged mice.	(70)
Dicam	Dicam expressed in quiescent and proliferating chondrocytes of the growth plate enhances growth plate chondrocyte proliferation and maturation with enhanced Ihh and PTHrP signaling.	(71)
NG2	NG2/CSPG4 affects chondrocyte differentiation by ERK1/2 signaling	(72)
TRPV4	TRPV4 mutations alter BMP signaling in chondrocytes and prevent proper chondrocyte hypertrophy	(73)
PC2	Polycystin is required for mechanical transduction of chondrocytes	(74)
HDAC6	Overexpression of HDAC6 causes mitochondrial dysfunction and promotes reactive oxygen species production, leading to ECM degradation.	(75)
Arl13b	Associated with altered hedgehog signaling in chondrocytes.	(76, 77)
HIF-2α	Up-regulation of HIF-2 α expression increases the expression of multiple catabolic genes and promotes ECM degradation in articular cartilage.	(78)
Gpr161	Knockdown of Gpr161 affected endochondral bone formation and prevented periarticular chondrocytes from differentiating into columnar chondrocytes while causing a lack of Ihh signaling.	(79)
Piezo1	Overexpression of Piezo1 in osteoarthritic cartilage and cultured chondrocytes under shear stress and Piezo1 silencing inhibits nuclear translocation of YAP and down-regulates MMP13 and ADAMTS5 expression Activation of Piezo1 promotes mechanically-induced cartilage degradation through the YAP-MMP13/ADAMTS5 signaling pathway.	(80)

THM2, modifier of Hedgehog signaling; PTHrP, Recombinant Parathyroid Hormone Related Protein; GRK2, G protein-coupled receptor kinase 2; Crocc2, encodes a key gene of the ciliary rootlets; FGF, Fibroblast Growth Factor; Dicam, Proteins localized to primary cilia; TRPV4, nonselective cation channel; PC2, tubulin deacetylase; HDAC6, a tubulin deacetylase; Arl13b, key regulator of the ciliary trafficking; HIF-2α, DNA-binding transcription factor; Gpr161, orphan G-proteincoupled receptor.

In addition, due to the unique composition of cartilage, its restoration following an injury proves highly challenging. Recently,Tao (81) found that activation of ciliated Hh signaling by Smoothened agonists (SAG) when the growth plate is damaged significantly accelerates growth plate repair after injury, and his further study found that chondrocytes in the resting and proliferating zones are dynamically ciliated during growth plate repair. Furthermore, conditional deletion of the cilia core gene Ift140 in cartilage disrupts cilia-mediated Hh signaling in the growth plate. These results show that ciliary Hh signaling by chondrocytes in the growth plate coordinates the cartilage regeneration response to GP injury. Additionally, primary cilia have also been reported to be involved in mechanical stress-mediated cartilage repair via the ERK/mTOR axis interacting with autophagy (82).

### 2.4 The role of primary cilia in fracture healing

Fracture healing is a significant clinical concern, and research has indicated that primary cilia and the ciliary transport system play a crucial role in the process of fracture healing. Through the targeted disruption of IFT88 in Prx1-Cre mice, Moore (65) observed that mice lacking primary cilia exhibited abnormally enlarged calluses, a substantial decrease in bone formation, and the presence of persistent cartilage nodules. Analysis of mRNA expression in early cartilage injury tissue revealed downregulation of osteogenesis, Hh signaling, and Wnt signaling, along with an upregulation of chondrogenesis and angiogenesis. Functionally, the deficiency of primary cilia resulted in delayed bone healing. Liu (83) conducted an investigation on the involvement of IFT80 protein in fracture healing, utilizing tamoxifen-induced Col2α1-CreER mice. The study revealed that Col2α1-creIFT80f/f mice displayed smaller fracture calluses compared to IFT80f/f mice. Furthermore, the Col2α1-creIFT80f/f mice exhibited low-density/porous woven bone tissue with significantly reduced bone volume, number of trabeculae, trabeculae thickness, and trabeculae spacing compared to the control mice. These findings indicate that the deletion of IFT80 led to a notable downregulation in the expression of angiogenic markers such as VEGF, PDGF, and angiopoietin, resulting in impaired vascularization of the fracture callus. From a mechanistic perspective, IFT80 deficiency reduces chondrocyte proliferation, cilia assembly and cartilage gene expression and differentiation by inhibiting TGF-βI and TGF-βR expression and Smad2/3 phosphorylation in fracture calluses, thereby downregulating the TGF-β signaling pathway.

In addition, primary cilia serve as central hubs for signaling and can directly facilitate signaling pathways such as Hh, Wnt, and TGFβ. Serowoky (84) provided evidence demonstrating the essential role of the Hh signaling pathway in fracture healing. Research studies have indicated that Hh signaling is initially downregulated during the early phase of fracture repair, but becomes upregulated in the periosteum during the later stages of the healing process. Research studies have indicated that Hh signaling is initially downregulated during the early phase of fracture repair, but becomes upregulated in the periosteum during the later stages of the healing process. However, Moore’s findings, using lineage tracing of prx+ cells, showed that the increased Hh signaling did not align with Prx1-expressing cells and their descendants in the corpus callosum and new bone tissue (65). These results suggest that the regulation of Hh signaling in the absence of cilia is non-autonomous. Interestingly, in contrast to previous studies that reported an important role for IHH in the healing of mandibular fractures in zebrafish, Serowoky found a large role for SHH in rib fractures in mice, His findings indicated an increased expression of SHH in the formation of intrachondral and intramembranous bone at the fracture site (84). Notably, the conditional knockdown of Shh during the late stage of fracture severely hindered the formation of cartilage callus and impeded fracture healing. Moreover, the upregulation of Shh expression and the requirement for Shh and Smo seemed to be specific to massive fracture injuries, as they were not observed in small fracture healing scenarios. Intriguingly, it has been observed that Shh cKO mice do not depend on the Hh mechanism for repair near the cut end, but instead rely on Shh for fracture healing in the central region of the affected site (84). Additionally, the classical Wnt/β-catenin signaling pathway is upregulated during fracture healing and contributes to the promotion of repair (85). Moore’s research demonstrated that the inhibition of Wnt led to an enlarged corpus callosum in wild-type mice, which aligns with the phenotype observed in mice lacking cilia (65). Furthermore, it has been discovered that TGF-β signaling is upregulated during the initial phases of fracture healing (86). This signaling pathway promotes fracture repair by binding to cell surface receptors and triggering the phosphorylation of Smad proteins, particularly Smad2/3.The activation of Smad2/3 has various effects on fracture healing. It enhances the proliferation and differentiation of mesenchymal stem cells, stimulates chondrogenesis, increases the production of matrix proteins, and promotes neointima formation in fracture calluses (87). Additionally, bone morphogenetic protein-2 (BMP-2), a member of the TGF-β superfamily, plays a significant role in activating osteoblasts and promoting bone formation. Activation of BMP-2 phosphorylates Smad and facilitates its translocation to the nucleus, activating p38 signaling. This, in turn, upregulates the expression of Runt-associated transcription factor 2 (RUNX2), which further promotes the process of fracture healing (88).

In recent years, there has been growing interest in understanding the association between diabetes and fracture risk. Numerous studies have highlighted that type 1 diabetes mellitus (T1DM) can impede fracture healing by impacting osteoblasts and their precursor cells (89, 90). Recently, Liu conducted a study to examine the impact of T1DM on primary cilia using a mouse model of streptozotocin-induced diabetes. The study also investigated the influence of primary cilia on fracture healing by utilizing osteoblast cell lines with IFT80 deletion through the use of OSX;IFT80f/f mice. The findings of the study demonstrated that diabetes hindered the expression of cilia genes and impeded the formation of primary cilia to a similar degree as observed in normoglycemic mice with IFT80 deficiency. Furthermore, both diabetic mice and normoglycemic mice with osteoblast cilia deletion exhibit delayed fracture healing significantly reduced bone density and mechanical strength, and decreased vascular formation. These results confirm the importance of primary cilia in bone regeneration during the fracture healing process (83). Indeed, a recent report by Chinipardaz highlighted that diabetes leads to the loss of primary cilia in chondrocytes and osteoblasts. Furthermore, the study revealed that in the diabetic environment, FOXO1 plays a significant role in reducing cilia formation by inhibiting the expression of cilia genes. As a result, this disruption in primary cilia contributes to the delay in the fracture healing process (24). This finding sheds new light on the critical role of primary cilia in the context of fracture healing and offers valuable insights into the mechanisms involved.

### 2.5 On the crosstalk between primary cilia and integrins in bone

Integrins are heterodimeric transmembrane receptors composed of α and β subunits that link the extracellular matrix to the intracellular skeleton at different focal adhesion (FA) sites (91). Integrins are linked to primary cilia via the actin cytoskeleton, recent research has revealed their direct involvement in linking the basal body of primary cilia to the actin cytoskeleton in a specific structure of cilia adhesion (92). In fact, cilia have long been reported to coordinate integrin signaling (93), however, recent studies have found that integrins can also influence cilia formation. A gene-wide screen reported a key role for integrin β1 in primary ciliogenesis, Failler (94) silenced TLN1, LAMB4, FERMT2, VTN, ITGB1 and Rac1 in Hs578T cells and indicated that ablation with shRNAs significantly increased the number of ciliated cells, with ablation of TLN1, FERMT2 and VTN having the most profound effect, resulting in a population of over 30% of cells assembling cilia. Interestingly, the knockdown of ITGB1 or Rac1 produced similar results, confirming that integrins are associated with ciliogenesis. Previous studies reported that integrins and can activate the mitogen-activated protein kinase (MAPK) pathway and that MAPK stimulates F-actin rearrangement and cilia lengthening by phosphorylating IFT motor proteins (95). In addition, recent studies have found that integrins and primary cilia interact in bone metabolism. Geoghegan (92) found that estrogen withdrawal associated with postmenopausal osteoporosis leads to disruption of αvβ3 integrins and reduced actin contractility, which in turn leads to cilia prolongation resulting in Hh pathway activation and paracrine signaling in osteoclasts. Stam (96)investigated the role of integrin α1β1 in primary cilia and the F-actin cytoskeleton in response to osteoarthritic mediators, measuring primary cilia length and F-actin peak numbers in isolated wild-type and itga1-null chondrocytes, and showed that integrin α1β1 is required to mediate cilia lengthening and the formation of F-actin peaks in response to hypotonic stress and IL-1. In addition, integrins and primary cilia, together with other mechanosensing systems such as polycystins and TAZ, may also generate multifaceted mechanosensing networks in the skeleton. This suggests that mechanosensing and mechanotransduction pathways in bone are fundamental to understanding bone physiology and therefore the specific molecular mechanisms of mechanosensors in the bone microenvironment still need to be further explored in subsequent work.

## 3 Conclusions and perspectives

Recent advances in understanding the role of primary cilia in bone metabolism are reviewed in this paper. Current research on primary cilia has focused on the basic function of primary cilia, ciliopathy and primary ciliary signaling pathways, and the crosstalk between primary cilia and other receptors and ciliary proteins is largely unexplored. Furthermore, it has been observed that primary cilia undergo elongation and curvature in response to mechanical forces. However, no studies have yet investigated the impact of cilia elongation, bending amplitude, and frequency on signaling pathways and the development of bone metabolic disorders.

In conclusion, this paper reaffirms the importance of primary cilia in bone development and emphasizes the necessity for continued research on primary cilia-associated proteins and signaling pathways. By targeting these aspects, more advanced treatments for ciliopathies can be developed, offering enhanced efficacy in the future.

## Author contributions

FFL: Conceptualization, Investigation, Writing – original draft. HL: Investigation, Writing – review & editing. YWM: Writing – review & editing. RZ: Writing – review & editing. WW: Conceptualization, Project administration, Funding acquisition.

## References

[1] Zheng N Miao YT Zhang X Huang MZ Jahangir M Luo S. Primary cilia–associated protein IFT172 in ciliopathies. Front Cell Dev Biol (2023) 11:30. doi: 10.3389/fcell.2023.1074880 PMC9887189 36733456

[2] Satir P Pedersen LB Christensen ST. The primary cilium at a glance. J Cell Sci (2010) 123(4):499–503. doi: 10.1242/jcs.050377 20144997 PMC2818190

[3] Sorokin S. Centrioles and the formation of rudimentary cilia by fibroblasts and smooth muscle cells. J Cell Biol (1962) 15(2):363–77. doi: 10.1083/jcb.15.2.363 PMC2106144 13978319

[4] Pedersen LB Rosenbaum JL. Intraflagellar transport (IFT) role in ciliary assembly, resorption and signalling. Curr Top Dev Biol (2008) 85:23–61. doi: 10.1016/S0070-2153(08)00802-8 19147001

[5] Leroux MR. Taking vesicular transport to the cilium. Cell (2007) 129(6):1041–3. doi: 10.1016/j.cell.2007.05.049 17574016

[6] Avasthi P Marshall W. Ciliary secretion: switching the cellular antenna to 'transmit'. Curr Biol (2013) 23(11):R471–3. doi: 10.1016/j.cub.2013.04.056 23743409

[7] Knödler A Feng S Zhang J Zhang X Das A Peränen J. Coordination of Rab8 and Rab11 in primary ciliogenesis. Proc Natl Acad Sci U S A (2010) 107(14):6346–51. doi: 10.1073/pnas.1002401107 PMC2851980 20308558

[8] Satir P Christensen ST. Overview of structure and function of mammalian cilia. Annu Rev Physiol (2007) 69:377–400. doi: 10.1146/annurev.physiol.69.040705.141236 17009929

[9] Sun S Fisher RL Bowser SS Pentecost BT Sui H. Three–dimensional architecture of epithelial primary cilia. Proc Natl Acad Sci U S A (2019) 116(19):9370–9. doi: 10.1073/pnas.1821064116 PMC6511023 31004057

[10] Kiesel P Alvarez Viar G Tsoy N Maraspini R Gorilak P Varga V. The molecular structure of mammalian primary cilia revealed by cryo–electron tomography. Nat Struct Mol Biol (2020) 27(12):1115–24. doi: 10.1038/s41594-020-0507-4 PMC7610599 32989303

[11] Rosenbaum JL Cole DG Diener DR. Intraflagellar transport: the eyes have it. J Cell Biol (1999) 144(3):385–8. doi: 10.1083/jcb.144.3.385 PMC2132910 9971734

[12] Hiyamizu S Qiu H Vuolo L Stevenson NL Shak C Heesom KJ. Multiple interactions of the dynein–2 complex with the IFT–B complex are required for effective intraflagellar transport. J Cell Sci (2023) 136(5):jcs260462. doi: 10.1242/jcs.260462 36632779 PMC10110421

[13] Hu Q Milenkovic L Jin H Scott MP Nachury MV Spiliotis ET. A septin diffusion barrier at the base of the primary cilium maintains ciliary membrane protein distribution. Science (2010) 329(5990):436–9. doi: 10.1126/science.1191054 PMC3092790 20558667

[14] Ishikawa H Marshall WF. Ciliogenesis: building the cell's antenna. Nat Rev Mol Cell Biol (2011) 12(4):222–34. doi: 10.1038/nrm3085 21427764

[15] Tao F Jiang T Tao H Cao H Xiang W. Primary cilia: Versatile regulator in cartilage development. Cell Prolif (2020) 53(3):e12765. doi: 10.1111/cpr.12765 32034931 PMC7106963

[16] Zhou H Wu S Ling H Zhang C Kong Y. Primary cilia: A cellular regulator of articular cartilage degeneration. Stem Cells Int (2022) 2022:2560441. doi: 10.1155/2022/2560441 36193252 PMC9525753

[17] Goetz SC Anderson KV. The primary cilium: a signalling centre during vertebrate development. Nat Rev Genet (2010) 11(5):331–44. doi: 10.1038/nrg2774 PMC3121168 20395968

[18] Ishikawa T Ueno H Omori T Kikuchi K. Cilia and centrosomes: Ultrastructural and mechanical perspectives. Semin Cell Dev Biol (2021) 110:61–9. doi: 10.1016/j.semcdb.2020.03.007 32307225

[19] Moruzzi N Leibiger B Barker CJ Leibiger IB Berggren PO. Novel aspects of intra–islet communication: Primary cilia and filopodia. Adv Biol Regul (2022) 87:100919. doi: 10.1016/j.jbior.2022.100919 36266190

[20] Nishimura Y Kasahara K Shiromizu T Watanabe M Inagaki M. Primary cilia as signaling hubs in health and disease. Adv Sci (2018) 6(1):1801138. doi: 10.1002/advs.201801138 PMC6325590 30643718

[21] McConnachie DJ Stow JL Mallett AJ. Ciliopathies and the kidney: A review. Am J Kidney Dis (2021) 77(3):410–9. doi: 10.1053/j.ajkd.2020.08.012 33039432

[22] Wang L You X Zhang L Zhang C Zou W. Mechanical regulation of bone remodeling. Bone Res (2022) 10(1):16. doi: 10.1038/s41413-022-00190-4 35181672 PMC8857305

[23] Guleria VS Parit R Quadri N Das R Upadhyai P. The intraflagellar transport protein IFT52 associated with short–rib thoracic dysplasia is essential for ciliary function in osteogenic differentiation in *vitro* and for sensory perception in Drosophila. Exp Cell Res (2022) 418(2):113273. doi: 10.1016/j.yexcr.2022.113273 35839863

[24] Chinipardaz Z Liu M Graves DT Yang S. Role of primary cilia in bone and cartilage. J Dent Res (2022) 101(3):253–60. doi: 10.1177/00220345211046606 PMC8866845 34743626

[25] Liu J Leng FF Gao YH He WF Wang JF Xian CJ. Protection of primary cilia is an effective countermeasure against the impairment of osteoblast function induced by simulated microgravity. J Cell Mol Med (2023) 27(1):36–51. doi: 10.1111/jcmm.17628 36512344 PMC9806295

[26] Saito M Hirano M Izumi T Mori Y Ito K Saitoh Y. Cytoskeletal protein 4.1G is essential for the primary ciliogenesis and osteoblast differentiation in bone formation. Int J Mol Sci (2022) 23(4):2094. doi: 10.3390/ijms23042094 35216233 PMC8878336

[27] Tummala P Arnsdorf EJ Jacobs CR. The role of primary cilia in mesenchymal stem cell differentiation: A pivotal switch in guiding lineage commitment. Cell Mol Bioeng (2010) 3(3):207–12. doi: 10.1007/s12195-010-0127-x PMC2930791 20823950

[28] Bodle JC Rubenstein CD Phillips ME Bernacki SH Qi J Banes AJ. Primary cilia: the chemical antenna regulating human adipose–derived stem cell osteogenesis. PloS One (2013) 8(5):e62554. doi: 10.1371/journal.pone.0062554 23690943 PMC3656889

[29] Izu Y Sun M Zwolanek D Veit G Williams V Cha B. Type XII collagen regulates osteoblast polarity and communication during bone formation. J Cell Biol (2011) 193(6):1115–30. doi: 10.1083/jcb.201010010 PMC3115787 21670218

[30] Shimada IS Kato Y. Ciliary signaling in stem cells in health and disease: Hedgehog pathway and beyond. Semin Cell Dev Biol (2022) 129:115–25. doi: 10.1016/j.semcdb.2022.04.011 35466055

[31] Moore ER Zhu YX Ryu HS Jacobs CR. Correction to: Periosteal progenitors contribute to load–induced bone formation in adult mice and require primary cilia to sense mechanical stimulation. Stem Cell Res Ther (2018) 9(1):229. doi: 10.1186/s13287-018-0975-1 29996901 PMC6042447

[32] Zhou J Gao YH Zhu BY He WF Wang G Xian CJ. The frequency window effect of sinusoidal electromagnetic fields in promoting osteogenic differentiation and bone formation involves extension of osteoblastic primary cilia and activation of protein kinase A. Cell Biol Int (2021) 45(8):1685–97. doi: 10.1002/cbin.11606 33811714

[33] He WF Qin R Gao YH Zhou J Wei JJ Liu J. The interdependent relationship between the nitric oxide signaling pathway and primary cilia in pulse electromagnetic field–stimulated osteoblastic differentiation. FASEB J (2022) 36(6):e22376. doi: 10.1096/fj.202101577RR 35616355

[34] Zheng L Zhou D Ju F Liu Z Yan C Dong Z. Oscillating fluid flow activated osteocyte lysate–based hydrogel for regulating osteoblast/osteoclast homeostasis to enhance bone repair. Adv Sci (Weinh) (2023) 10(15):e2204592. doi: 10.1002/advs.202204592 37017573 PMC10214251

[35] Martín–Guerrero E Tirado–Cabrera I Buendía I Alonso V Gortázar AR Ardura JA. Primary cilia mediate parathyroid hormone receptor type 1 osteogenic actions in osteocytes and osteoblasts via Gli activation. J Cell Physiol (2020) 235(10):7356–69. doi: 10.1002/jcp.29636 32039485

[36] Spasic M Duffy MP Jacobs CR. Fenoldopam sensitizes primary cilia–mediated mechanosensing to promote osteogenic intercellular signaling and whole bone adaptation. J Bone Miner Res (2022) 37(5):972–82. doi: 10.1002/jbmr.4536 PMC9098671 35230705

[37] Yuan X Yang S. Primary cilia and intraflagellar transport proteins in bone and cartilage. J Dent Res (2016) 95(12):1341–9. doi: 10.1177/0022034516652383 PMC5076748 27250654

[38] Zhang C Zhang S Sun Y. Expression of IFT140 during bone development. J Histochem Cytochem (2019) 67(10):723–34. doi: 10.1369/0022155419859357 PMC6764064 31238004

[39] Tao D Xue H Zhang C Li G Sun Y. The role of IFT140 in osteogenesis of adult mice long bone. J Histochem Cytochem (2019) 67(8):601–11. doi: 10.1369/0022155419847188 PMC6669857 31034313

[40] Chen Y Fan Q Zhang H Tao D Wang Y Yue R. Lineage tracing of cells expressing the ciliary gene IFT140 during bone development. Dev Dyn (2021) 250(4):574–83. doi: 10.1002/dvdy.266 33095947

[41] Chen Q Shou P Zheng C Jiang M Cao G Yang Q. Fate decision of mesenchymal stem cells: adipocytes or osteoblasts? Cell Death Differ (2016) 23(7):1128–39. doi: 10.1038/cdd.2015.168 PMC4946886 26868907

[42] Li Y Yang S Liu Y Qin L Yang S. IFT20 governs mesenchymal stem cell fate through positively regulating TGF–β–Smad2/3–Glut1 signaling mediated glucose metabolism. Redox Biol (2022) 54:102373. doi: 10.1016/j.redox.2022.102373 35751983 PMC9243161

[43] Cavalcanti DP Huber C Sang KH Baujat G Collins F Delezoide AL. Mutation in IFT80 in a fetus with the phenotype of Verma–Naumoff provides molecular evidence for Jeune–Verma–Naumoff dysplasia spectrum. J Med Genet (2011) 48(2):88–92. doi: 10.1136/jmg.2009.069468 19648123

[44] Haycraft CJ Zhang Q Song B Jackson WS Detloff PJ Serra R. Intraflagellar transport is essential for endochondral bone formation. Development (2007) 134(2):307–16. doi: 10.1242/dev.02732 17166921

[45] Rix S Calmont A Scambler PJ Beales PL. An Ift80 mouse model of short rib polydactyly syndromes shows defects in hedgehog signalling without loss or malformation of cilia. Hum Mol Genet (2011) 20(7):1306–14. doi: 10.1093/hmg/ddr013 PMC3049354 21227999

[46] Allard BA Wang W Pottorf TS Mumtaz H Jack BM Wang HH. Thm2 interacts with paralog, Thm1, and sensitizes to Hedgehog signaling in postnatal skeletogenesis. Cell Mol Life Sci (2021) 78(7):3743–62. doi: 10.1007/s00018-021-03806-w PMC9278483 33683377

[47] Xiao Z Zhang S Magenheimer BS Luo J Quarles LD. Polycystin–1 regulates skeletogenesis through stimulation of the osteoblast–specific transcription factor RUNX2–II. J Biol Chem (2008) 283(18):12624–34. doi: 10.1074/jbc.M710407200 PMC2335361 18321855

[48] Xiao Z Dallas M Qiu N Nicolella D Cao L Johnson M. Conditional deletion of Pkd1 in osteocytes disrupts skeletal mechanosensing in mice. FASEB J (2011) 25(7):2418–32. doi: 10.1096/fj.10-180299 PMC3219213 21454365

[49] Xiao Z Cao L Liang Y Huang J Stern AR Dallas M. Osteoblast–specific deletion of Pkd2 leads to low–turnover osteopenia and reduced bone marrow adiposity. PloS One (2014) 9(12):e114198. doi: 10.1371/journal.pone.0114198 25464512 PMC4252138

[50] Koyama E Young B Nagayama M Shibukawa Y Enomoto-Iwamoto M Iwamoto M. Conditional Kif3a ablation causes abnormal hedgehog signaling topography, growth plate dysfunction, and excessive bone and cartilage formation during mouse skeletogenesis. Development (2007) 134(11):2159–69. doi: 10.1242/dev.001586 PMC2776720 17507416

[51] Temiyasathit S Tang WJ Leucht P Anderson CT Monica SD Castillo AB. Mechanosensing by the primary cilium: deletion of Kif3A reduces bone formation due to loading. PloS One (2012) 7(3):e33368. doi: 10.1371/journal.pone.0033368 22428034 PMC3299788

[52] Qiu N Xiao Z Cao L Buechel MM David V Roan E. Disruption of Kif3a in osteoblasts results in defective bone formation and osteopenia. J Cell Sci (2012) 125(Pt 8):1945–57. doi: 10.1242/jcs.095893 PMC3360919 22357948

[53] Xiao Z Zhang S Mahlios J Zhou G Magenheimer BS Guo D. Cilia–like structures and polycystin–1 in osteoblasts/osteocytes and associated abnormalities in skeletogenesis and Runx2 expression. J Biol Chem (2006) 281(41):30884–95. doi: 10.1074/jbc.M604772200 PMC1797154 16905538

[54] Lim J Li X Yuan X Yang S Han L Yang S. Primary cilia control cell alignment and patterning in bone development via ceramide–PKCζ–β–catenin signaling. Commun Biol (2020) 3(1):45. doi: 10.1038/s42003-020-0767-x 31988398 PMC6985158

[55] Park I Lee HK Kim C Ismail T Kim YK Park JW. IFT46 plays crucial roles in craniofacial and cilia development. Biochem Biophys Res Commun (2016) 477(3):419–25. doi: 10.1016/j.bbrc.2016.06.083 27320864

[56] Hao X Wang D Yan Z Ding Y Zhang J Liu J. Bone deterioration in response to chronic high–altitude hypoxia is attenuated by a pulsed electromagnetic field via the primary cilium/HIF–1α Axis. J Bone Miner Res (2023) 38(4):597–614. doi: 10.1002/jbmr.4772 36680558

[57] Guo J Ren R Sun K Yao X Lin J Wang G. PERK controls bone homeostasis through the regulation of osteoclast differentiation and function. Cell Death Dis (2020) 11(10):847. doi: 10.1038/s41419-020-03046-z 33051453 PMC7554039

[58] Sutton MM Duffy MP Verbruggen SW Jacobs CR. Osteoclastogenesis requires primary cilia disassembly and can be inhibited by promoting primary cilia formation pharmacologically [published online ahead of print, 2023 May 22]. Cells Tissues Organs (2023). doi: 10.1159/000531098 PMC10863750 37231815

[59] Pi Y Liang H Yu Q Yin Y Xu H Lei Y. Low–frequency pulsed electromagnetic field inhibits RANKL–induced osteoclastic differentiation in RAW264.7 cells by scavenging reactive oxygen species. Mol Med Rep (2019) 19(5):4129–36. doi: 10.3892/mmr.2019.10079 PMC6470919 30942408

[60] Lei Y Su J Xu H Yu Q Zhao M Tian J. Pulsed electromagnetic fields inhibit osteoclast differentiation in RAW264.7 macrophages via suppression of the protein kinase B/mammalian target of rapamycin signaling pathway. Mol Med Rep (2018) 18(1):447–54. doi: 10.3892/mmr.2018.8999 29749519

[61] Wang P Tang C Wu J Yang Y Yan Z Liu X. Pulsed electromagnetic fields regulate osteocyte apoptosis, RANKL/OPG expression, and its control of osteoclastogenesis depending on the presence of primary cilia. J Cell Physiol (2019) 234(7):10588–601. doi: 10.1002/jcp.27734 30422320

[62] Tirado–Cabrera I Martin–Guerrero E Heredero–Jimenez S Ardura JA Gortázar AR. PTH1R translocation to primary cilia in mechanically–stimulated ostecytes prevents osteoclast formation via regulation of CXCL5 and IL–6 secretion. J Cell Physiol (2022) 237(10):3927–43. doi: 10.1002/jcp.30849 PMC9804361 35933642

[63] Wu Q Zhou X Huang D Ji Y Kang F. IL–6 enhances osteocyte–mediated osteoclastogenesis by promoting JAK2 and RANKL activity *in vitro* . Cell Physiol Biochem (2017) 41(4):1360–9. doi: 10.1159/000465455 28278513

[64] Deepak V Yang ST Li Z Li X Ng A Xu D. IFT80 negatively regulates osteoclast differentiation via association with Cbl–b to disrupt TRAF6 stabilization and activation [published correction appears in Proc Natl Acad Sci U S A. 2022 Sep 13,119(37):e2212944119]. Proc Natl Acad Sci U S A (2022) 119(26):e2201490119. doi: 10.1073/pnas.2201490119 35733270 PMC9245634

[65] Moore ER Mathews OA Yao Y Yang Y. Prx1–expressing cells contributing to fracture repair require primary cilia for complete healing in mice. Bone (2021) 143:115738. doi: 10.1016/j.bone.2020.115738 33188955 PMC7769995

[66] Salhotra A Shah HN Levi B Longaker MT. Mechanisms of bone development and repair. Nat Rev Mol Cell Biol (2020) 21(11):696–711. doi: 10.1038/s41580-020-00279-w 32901139 PMC7699981

[67] Hallett SA Matsushita Y Ono W Sakagami N Mizuhashi K Tokavanich N. Chondrocytes in the resting zone of the growth plate are maintained in a Wnt–inhibitory environment. Elife (2021) 10:e64513. doi: 10.7554/eLife.64513 34309509 PMC8313235

[68] Bosakova M Abraham SP Nita A Hruba E Buchtova M Taylor SP. Mutations in GRK2 cause Jeune syndrome by impairing Hedgehog and canonical Wnt signaling. EMBO Mol Med (2020) 12(11):e11739. doi: 10.15252/emmm.201911739 33200460 PMC7645380

[69] Packard MC Gilbert MC Tetrault E Albertson RC. Zebrafish crocc2 mutants exhibit divergent craniofacial shape, misregulated variability, and aberrant cartilage morphogenesis. Dev Dyn (2023) 252(7):1026–45. doi: 10.1002/dvdy.591 PMC10524572 37032317

[70] Dutra EH Chen PJ Kalajzic Z Wadhwa S Hurley M Yadav S. and Receptors in Osteochondral Tissues of the Temporomandibular Joint in Young and Aging Mice [published online ahead of print, 2023 Apr 26]. Cartilage (2023):19476035231163691. doi: 10.1177/19476035231163691 37098717 PMC11368896

[71] Han S Park HR Lee EJ Jang JA Han MS Kim GW. Dicam promotes proliferation and maturation of chondrocyte through Indian hedgehog signaling in primary cilia. Osteoarthritis Cartilage (2018) 26(7):945–53. doi: 10.1016/j.joca.2018.04.008 29702220

[72] Reed DA Zhao Y Bagheri Varzaneh M Shin JS Rozynek J Miloro M. NG2/CSPG4 regulates cartilage degeneration during TMJ osteoarthritis. Front Dent Med (2022) 3:1004942. doi: 10.3389/fdmed.2022.1004942 36685663 PMC9850834

[73] Dicks AR Maksaev GI Harissa Z Savadipour A Tang R Steward N. Skeletal dysplasia–causing TRPV4 mutations suppress the hypertrophic differentiation of human iPSC–derived chondrocytes. Elife (2023) 12:e71154. doi: 10.7554/eLife.71154 36810131 PMC9949800

[74] Thompson CL McFie M Chapple JP Beales P Knight MM. Polycystin–2 is required for chondrocyte mechanotransduction and traffics to the primary cilium in response to mechanical stimulation. Int J Mol Sci (2021) 22(9):4313. doi: 10.3390/ijms22094313 33919210 PMC8122406

[75] Zheng Y Chen Y Lu X Weng Q Dai G Yu Y. Inhibition of histone deacetylase 6 by tubastatin A attenuates the progress of osteoarthritis via improving mitochondrial function. Am J Pathol (2020) 190(12):2376–86. doi: 10.1016/j.ajpath.2020.08.013 32926854

[76] Thorpe SD Gambassi S Thompson CL Chandrakumar C Santucci A Knight MM. Reduced primary cilia length and altered Arl13b expression are associated with deregulated chondrocyte Hedgehog signaling in alkaptonuria. J Cell Physiol (2017) 232(9):2407–17. doi: 10.1002/jcp.25839 PMC5484994 28158906

[77] Li Y Ling K Hu J. The emerging role of Arf/Arl small GTPases in cilia and ciliopathies. J Cell Biochem (2012) 113(7):2201–7. doi: 10.1002/jcb.24116 PMC4133128 22389062

[78] Ito Y Matsuzaki T Ayabe F Mokuda S Kurimoto R Matsushima T. Both microRNA–455–5p and –3p repress hypoxia–inducible factor–2α expression and coordinately regulate cartilage homeostasis. Nat Commun (2021) 12(1):4148. doi: 10.1038/s41467-021-24460-7 34230481 PMC8260725

[79] Hwang SH White KA Somatilaka BN Shelton JM Richardson JA Mukhopadhyay S. The G protein–coupled receptor Gpr161 regulates forelimb formation, limb patterning and skeletal morphogenesis in a primary cilium–dependent manner. Development (2018) 145(1):dev154054. doi: 10.1242/dev.154054 29222391 PMC5825871

[80] Feng X Li S Wang S Meng Y Zheng S Liu C. Piezo1 mediates the degradation of cartilage extracellular matrix in malocclusion–induced TMJOA [published online ahead of print, 2023 May 15]. Oral Dis (2023). doi: 10.1111/odi.14615 37184045

[81] Tao D Zhang L Ding Y Tang N Xu X Li G. Primary cilia support cartilage regeneration after injury. Int J Oral Sci (2023) 15(1):22. doi: 10.1038/s41368-023-00223-6 37268650 PMC10238430

[82] Xiang W Jiang T Hao X Wang R Yao X Sun K. Primary cilia and autophagy interaction is involved in mechanical stress mediated cartilage development via ERK/mTOR axis. Life Sci (2019) 218:308–13. doi: 10.1016/j.lfs.2019.01.001 30610869

[83] Liu M Alharbi M Graves D Yang S. IFT80 is required for fracture healing through controlling the regulation of TGF–β Signaling in chondrocyte differentiation and function. J Bone Miner Res (2020) 35(3):571–82. doi: 10.1002/jbmr.3902 PMC7525768 31643106

[84] Serowoky MA Kuwahara ST Liu S Vakhshori V Lieberman JR Mariani FV. A murine model of large–scale bone regeneration reveals a selective requirement for Sonic Hedgehog. NPJ Regener Med (2022) 7(1):30. doi: 10.1038/s41536-022-00225-8 PMC9114339 35581202

[85] Houschyar KS Tapking C Borrelli MR Popp D Duscher D Maan ZN. Wnt pathway in bone repair and regeneration – what do we know so far. Front Cell Dev Biol (2019) 6:170. doi: 10.3389/fcell.2018.00170 30666305 PMC6330281

[86] Phillips AM. Overview of the fracture healing cascade. Injury (2005) 36 Suppl 3:S5–7. doi: 10.1016/j.injury.2005.07.027 16188551

[87] Poniatowski ŁA Wojdasiewicz P Gasik R Szukiewicz D. Transforming growth factor Beta family: insight into the role of growth factors in regulation of fracture healing biology and potential clinical applications. Mediators Inflamm (2015) 2015:137823. doi: 10.1155/2015/137823 25709154 PMC4325469

[88] Jun JY Kim JH Kim M Hong S Kim M Ryu GH. Persicae semen promotes bone union in rat fractures by stimulating osteoblastogenesis through BMP–2 and wnt signaling. Int J Mol Sci (2023) 24(8):7388. doi: 10.3390/ijms24087388 37108563 PMC10138545

[89] Napoli N Chandran M Pierroz DD Abrahamsen B Schwartz AV Ferrari SL. Mechanisms of diabetes mellitus–induced bone fragility. Nat Rev Endocrinol (2017) 13(4):208–19. doi: 10.1038/nrendo.2016.153 27658727

[90] Lecka–Czernik B. Diabetes, bone and glucose–lowering agents: basic biology. Diabetologia (2017) 60(7):1163–9. doi: 10.1007/s00125-017-4269-4 PMC5487688 28434032

[91] Barczyk M Carracedo S Gullberg D. Integrins. Cell Tissue Res (2010) 339(1):269–80. doi: 10.1007/s00441-009-0834-6 PMC2784866 19693543

[92] Geoghegan IP McNamara LM Hoey DA. Estrogen withdrawal alters cytoskeletal and primary ciliary dynamics resulting in increased Hedgehog and osteoclastogenic paracrine signalling in osteocytes. Sci Rep (2021) 11(1):9272. doi: 10.1038/s41598-021-88633-6 33927279 PMC8085225

[93] Pittman AE Solecki DJ. Cooperation between primary cilia signaling and integrin receptor extracellular matrix engagement regulates progenitor proliferation and neuronal differentiation in the developing cerebellum. Front Cell Dev Biol (2023) 11:1127638. doi: 10.3389/fcell.2023.1127638 36895790 PMC9990755

[94] Failler M Giro–Perafita A Owa M Srivastava S Yun C Kahler DJ. Whole–genome screen identifies diverse pathways that negatively regulate ciliogenesis. Mol Biol Cell (2021) 32(2):169–85. doi: 10.1091/mbc.E20-02-0111 PMC8120696 33206585

[95] Wang Y Ren Y Pan J. Regulation of flagellar assembly and length in *Chlamydomonas* by LF4, a MAPK–related kinase. FASEB J (2019) 33(5):6431–41. doi: 10.1096/fj.201802375RR 30794426

[96] Stam LB Clark AL. Chondrocyte primary cilia lengthening and shortening in response to mediators of osteoarthritis, a role for integrin α1β1 and focal adhesions. Osteoarthr Cartil Open (2023) 5(2):100357. doi: 10.1016/j.ocarto.2023.100357 37008821 PMC10063384

